# Supplementary material for: Analysis of the temporal and spatial evolution of China’s air passenger transportation and the impact of policies on passenger flows
Source: PLoS One. 2025 May 23;20(5):e0323482. doi: 10.1371/journal.pone.0323482 (PMC12101684; doi:10.1371/journal.pone.0323482)
Supplement: Attachment — Submitted filename: 2025326-Response to Reviewers.docx [file pone.0323482.s003.docx]

**Response to Reviewer #1**

**Response**:

We thank the referee very much for carefully reviewing our paper and providing the very nice and constructive suggestions, which help improve the quality of this paper even further. It is our pleasure. In this revision, we have followed the suggestions to revise the paper accordingly, with all the revisions highlighted with red color.

***1. I dont think the authors have treated the revision serisouly. Again, please search and learn how other fine studies' structure and writing styles and present your wok in a good and appropriate way. Both Abstract, Introduction and Literature review still needs a big improvement (Sttructure, languages and the academic way how you present them). Also, in literature review, do not just copy and paste how other scholars done their works, summarize them, compare them and present it in your own words. Figure quality still not bad, improve the quality to meet the standard and let readers easy to catch up.***

**Response to 1：**

Thank you for your valuable feedback. We have carefully reviewed relevant high-quality studies to refine the structure, language, and academic presentation of our manuscript. In particular, we have made comprehensive revisions to the abstract, introduction, and literature review to improve clarity and coherence.Additionally, we have reorganized the literature review to provide a more structured summary and comparison of previous studies, ensuring that our discussion is presented in our own words to maintain academic rigor.We appreciate your positive comments on the quality of our figures and tables.

**Response to Reviewer #2**

**Response**:

We thank the referee very much for the feedback and nice suggestions. In this revision, we have followed the suggestions to revise the paper accordingly, with all the revisions highlighted with red color.

***1.The innovations of this paper should be more clearly highlighted.***

**Response to 1：**

Thank you for your constructive suggestions. We have further emphasized the novelty of our study by explicitly outlining its unique contributions in the introduction (**pages 6, lines 113–124**).

***2.*** ***In the author information section, should the same organization be listed repeatedly? Please carefully verify the journal's formatting requirements.***

**Response to 2：**

We appreciate your attention to detail regarding formatting. We have carefully checked the journal’s formatting guidelines and have made the necessary adjustments to the author information section to ensure compliance with the journal’s requirements.

***3. It is suggested to indicate the enlarged area in the original image with a rectangle.***

**Response to 3：**

Following your recommendation, we have modified the figures by using rectangular markers to indicate magnified areas, thereby enhancing the clarity and readability of the visual representations.

***4. In section 2.3.1, it is recommended to place the introduction of the parameters after the appearance of the formula, and the same approach should be applied to the other formulas.***

**Response to 4：**

We also appreciate your meticulous review of the formula arrangement. In Section 2.3.1, we have revised the order of presentation, placing parameter descriptions after the equations for better logical flow. Additionally, we have applied this adjustment consistently throughout the manuscript to enhance readability and coherence.
