# Supplementary material for: Analysis of the temporal and spatial evolution of China’s air passenger transportation and the impact of policies on passenger flows
Source: PLoS One. 2025 May 23;20(5):e0323482. doi: 10.1371/journal.pone.0323482 (PMC12101684; doi:10.1371/journal.pone.0323482)
Supplement: Attachment — Submitted filename: 2025403-Response to Reviewers.docx [file pone.0323482.s004.docx]

**Academic Editor**

***1.*Please review your reference list to ensure that it is complete and correct. If you have cited papers that have been retracted, please include the rationale for doing so in the manuscript text, or remove these references and replace them with relevant current references. Any changes to the reference list should be mentioned in the rebuttal letter that accompanies your revised manuscript. If you need to cite a retracted article, indicate the article’s retracted status in the References list and also include a citation and full reference for the retraction notice.**

**Response to 1：**

Thank you for your valuable comments regarding our reference list. We have conducted a thorough review of all citations to ensure their completeness and accuracy, and can confirm that no retracted papers have been included. All references have been carefully formatted according to the journal's guidelines, with each entry verified for proper presentation. Should any further modifications be required prior to final publication, please do not hesitate to let us know - we would be happy to make any necessary adjustments.

**Response to Reviewer #1**

**Response**:

We thank the referee very much for carefully reviewing our paper and providing the very nice and constructive suggestions, which help improve the quality of this paper even further. It is our pleasure. In this revision, we have followed the suggestions to revise the paper accordingly, with all the revisions highlighted with red color.

***1.Writting a fine literature review, you need to use "your own" academic words to represent the literatrures you found related. Certainly, you can use one or two scholars' sentences, such as "A said...", "B used ... to do...". But you cannot use this structure everywhere in your literature review section.***

***Also, please make the legend in your figures more clearly to see.***

**Response to 1：**

We sincerely appreciate your constructive feedback on our manuscript. In response to your valuable suggestions, we have thoroughly revised the literature review section to present research findings in a more integrated manner while minimizing excessive direct citations. Additionally, we have enhanced all figure legends by incorporating them as high-resolution TIFF files to ensure optimal clarity when enlarged. We are grateful for your insightful comments and believe these revisions have significantly improved our manuscript. Should you have any further suggestions, we would be pleased to address them.

**Response to Reviewer #2**

**Response**:

We thank the referee very much for the feedback and nice suggestions.

***1.The author has addressed the previously raised concerns with appropriate revisions. The research content shows some relevance, and therefore, I recommend this manuscript for publication.***

**Response to 1：**

We sincerely appreciate your time and valuable feedback throughout the review process. Thank you for recognizing our efforts in addressing the previous concerns and for your positive recommendation regarding our manuscript's publication. We are grateful for your constructive comments, which have significantly contributed to improving the quality of our work.

Should there be any additional minor revisions required prior to final publication, please do not hesitate to let us know. We would be happy to make any necessary adjustments.

Thank you again for your thoughtful review and support.
